# Design of antipodal vivaldi antenna with patch and corrugations for 5G applications

**DOI:** 10.1016/j.mex.2024.102727

**Published:** 2024-04-27

**Authors:** Sumit Kumar, Amruta S. Dixit, Chandan Kumar Choubey

**Affiliations:** Symbiosis Institute of Technology, Pune Campus, Symbiosis International (Deemed University), Pune, 412115, Maharashtra, India

**Keywords:** Compact Size, 5 G applications, Corrugation, Antipodal Vivaldi Antenna (AVA) array, Parasitic patch, Methodology to design Antipodal Vivaldi antenna

## Abstract

A compact 1 × 4 antipodal Vivaldi antenna (AVA) array designed for 5 G applications is introduced in this study. An elliptical-shaped parasitic patch and corrugation are strategically employed to enhance gain and bandwidth, making it well-suited for 5 G applications. The resulting AVA array with corrugation and parasitic patch (AVA-PC) is designed and simulated on ANSYS HFSS, demonstrating a stable gain ranging from 10 dBi to 11.7 dBi over the frequency range of 23.45 GHz to 28.74 GHz. The antenna, with 25.8 mm x 22.4 mm x 0.5 mm dimensions, is implemented on Roger's RT/Duroid substrate 5880.

• Design uses an antipodal Vivaldi antenna to build a 1 × 4 AVA.
• The array employs corrugations and an elliptical patch as a performance enhancement technique.
• Simulated results confirm the designed antenna's practical utility for 5 G applications in a band of 23.45 GHz to 28.74 GHz.

## Graphical abstract

Attached as a separate file

**Figure:**
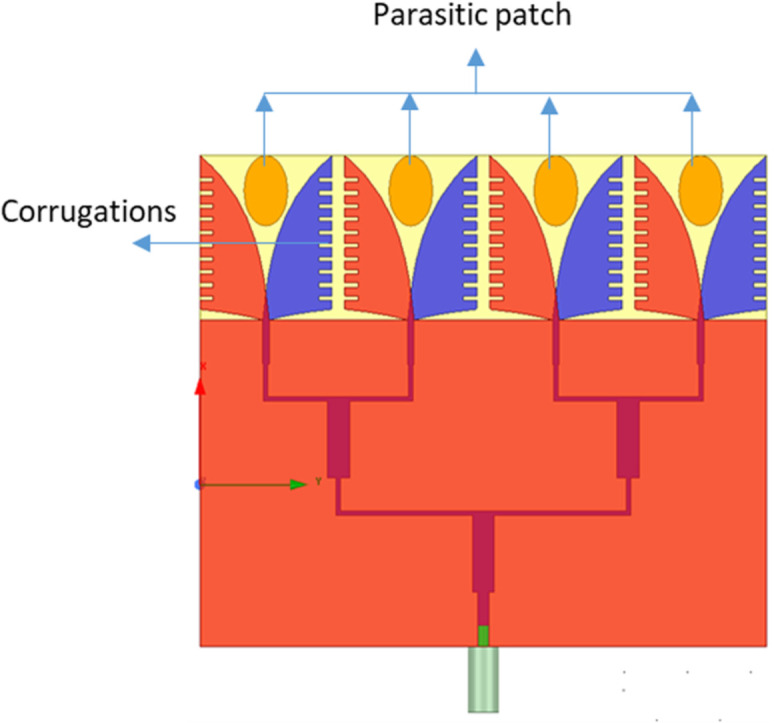

Specifications table

**Table:** 

Subject area:	Engineering
More specific subject area:	Antenna design
Name of your method:	Methodology to design Antipodal Vivaldi antenna
Name and reference of original method:	S. Zhu, H. Liu, Z. Chen and P. Wen, ``A Compact Gain-Enhanced Vivaldi Antenna Array With Suppressed Mutual Coupling for 5 G mmWave Application,'' in IEEE Antennas and Wireless Propagation Letters, vol. 17, no. 5, pp. 776–779, May 2018. https://doi.org/10.1109/LAWP.2018.2816038
Resource availability:	Method validation using ANSYS HFSS software

## Method details

Demand for wireless devices is increasing day by day. Consumers demand wireless devices with higher performance, like a higher operating range, battery life, ease of use, small in size, better and better performance, etc. Cellular phone evolution from 1 G to 4 G [1] greatly advances. These wireless technologies allow users to use the internet or other services whenever they want and from anywhere, anytime. Compared to 2 G and 3 G, 4 G has higher internet speed, better audio and video calling, higher performance, etc. Still, the present requirements of wireless devices are creating a burden on 4 G communication devices. The 5 G communication is the only panacea for current wireless communication devices. 5 G technology will perform much better than 4 G, with faster data rate (nearly Gbps), much better video calling, low latency, better quality of service (QoS), etc. Hence, a compact antenna with higher gain [[2], [3]], efficiency, stable radiation pattern, and more bandwidth for 5 G application is required. This gives rise to the origin of this proposal that an antenna should be designed to satisfy the requirements of 5 G communication devices. The 28 GHz and 38 GHz frequency bands have been designated by the International Telecommunication Union (ITU) [4] for 5 G applications [5], [6], [7]. This allocation is geared towards achieving superior data rates, enhanced spectrum efficiency, and an elevated quality of service, surpassing the capabilities of 4 G communication. Despite the progress in 5 G technology, challenges, such as path losses, persist, especially in millimeter-wave (mmWave) frequencies [8], [9], [10]. Overcoming these challenges requires antennas with increased efficiency and gain, making them well-suited for mmWave operation. The Antipodal Vivaldi Antenna (AVA) emerges as a promising solution, capable of operating at higher frequencies and delivering wide bandwidth [11], [12], [13], [14].

The AVA stands out as an optimal choice for 5 G devices due to its characteristics, including wide bandwidth, lightweight design, high efficiency, enhanced gain, ability to operate at high frequencies, and ease of fabrication. Various enhancement techniques have been explored to fine-tune Antipodal Vivaldi Antennas's (AVA) performance. These techniques encompass features such as corrugations, dielectric lenses, fractal AVA, parasitic patches, metamaterials, arrays, and the integration of multiple input and multiple output (MIMO) systems [15], [16], [17], [18]. In the literature, different shapes for parasitic patches [[19], [20]], including ellipses, diamonds, trapezoids, and circles, have been investigated. Notably, the elliptical shape has proven to be effective in enhancing the performance of AVA [21], [22], [23], [24]. This paper focuses on implementing an elliptical-shaped parasitic patch and corrugations to design a 1 × 4 AVA array to improve the AVA's bandwidth, gain, and efficiency.

**Fig. 1:**
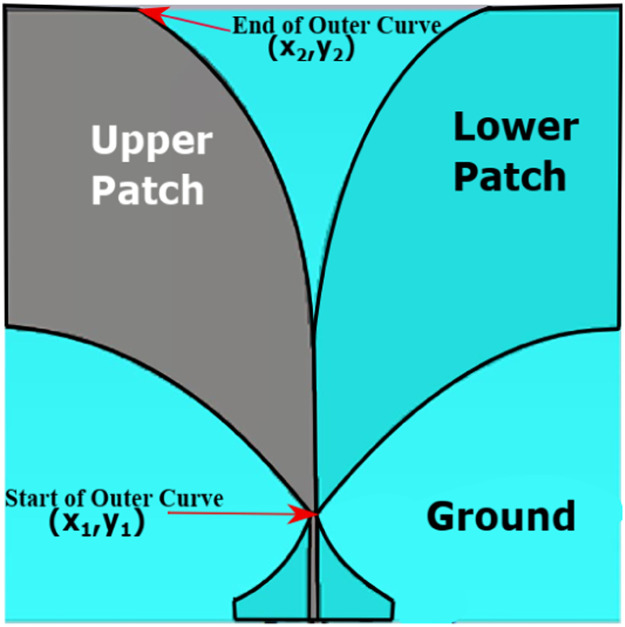
Antipodal Vivaldi antenna.

The antenna is constructed using the RT/Duroid 5880 substrate, known for its relative permittivity of 2.2 and loss tangent of 0.0009. Utilizing HFSS simulation software (version 2020 R2), the dimensions of the antenna are precisely optimized. In this configuration, the ground is formed by the AVA flare with the bottom exponential curve, whereas the other AVA flare represents the radiator. A microstrip line is utilized for feeding, optimized to achieve a 50Ω input impedance. The various design evolution steps are explained below :

Step 1: Design of Single Patch antenna: Fig. 1 shows the structure of the Antipodal Vivaldi antenna. The following set of equations gives the equation [25], [26], [27] of the tapered slot of the Antipodal Vivaldi antenna :

(1) $$Y=\pm \left(C_{1\;}e^{\mathrm{ax}}+C_{2\;}\right)$$

where C_1_ and C_2_ are outlined by the expression given by

(2) $$C_{1}=\frac{y_{2}-y_{1}}{e^{ax_{2}}-e^{ax_{1}}}$$

(3) $$C_{2}=\frac{e^{ax_{2}}y_{1}-e^{ax_{1}}y_{2}}{e^{ax_{2}}-e^{ax_{1}}}$$

These constants C_1_ and C_2_, along with the parameter ‘a,' govern the rate of increase of the exponential curve, which is visually depicted in Fig. 1. The exponential curve is characterized by its start and end points, denoted as x_1_, x_2_, y_1_, and y_2_, contributing to the overall shape and behavior of the curve.

**Fig. 2:**
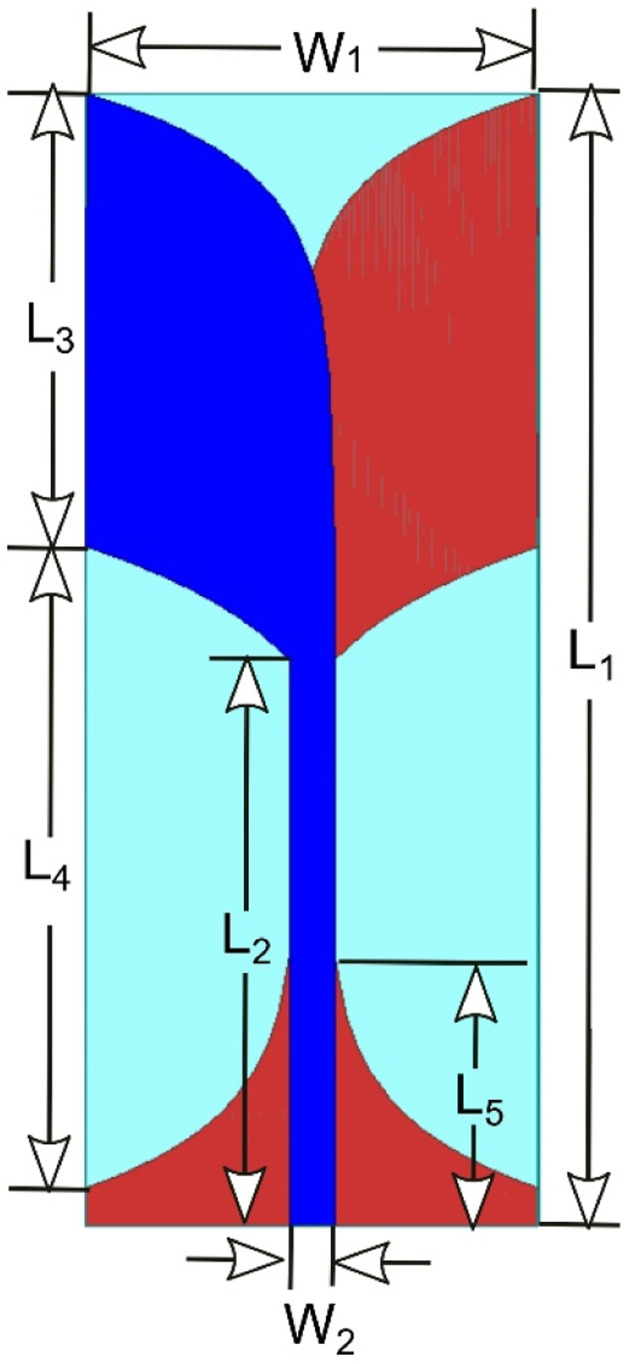
Design of Single Patch antenna.

Using the above equations of antipodal antenna, a single patch antenna is designed as shown in Fig. 2, with dimensions shown in Table 1. In this, the blue color represents the top patch (radiator). The red color represents the bottom patch (ground). L1 is the length of the substrate, W1 is the width of the substrate, L2 is the length of the feedline, W2 is the width of the feedline, L3, L4, and L5 represent the lengths of various parts of the antenna required to redesign the antenna by readers.

**Table 1:** Dimensions of CAVA.

Variable	Measurements(mm)	Parameter	Measurements(mm)
L_1_	15	W_1_	6
L_2_	7.5	W_2_	0.59
L_3_	6	L_4_	8.5
L_5_	3.6		

**Fig. 3:**
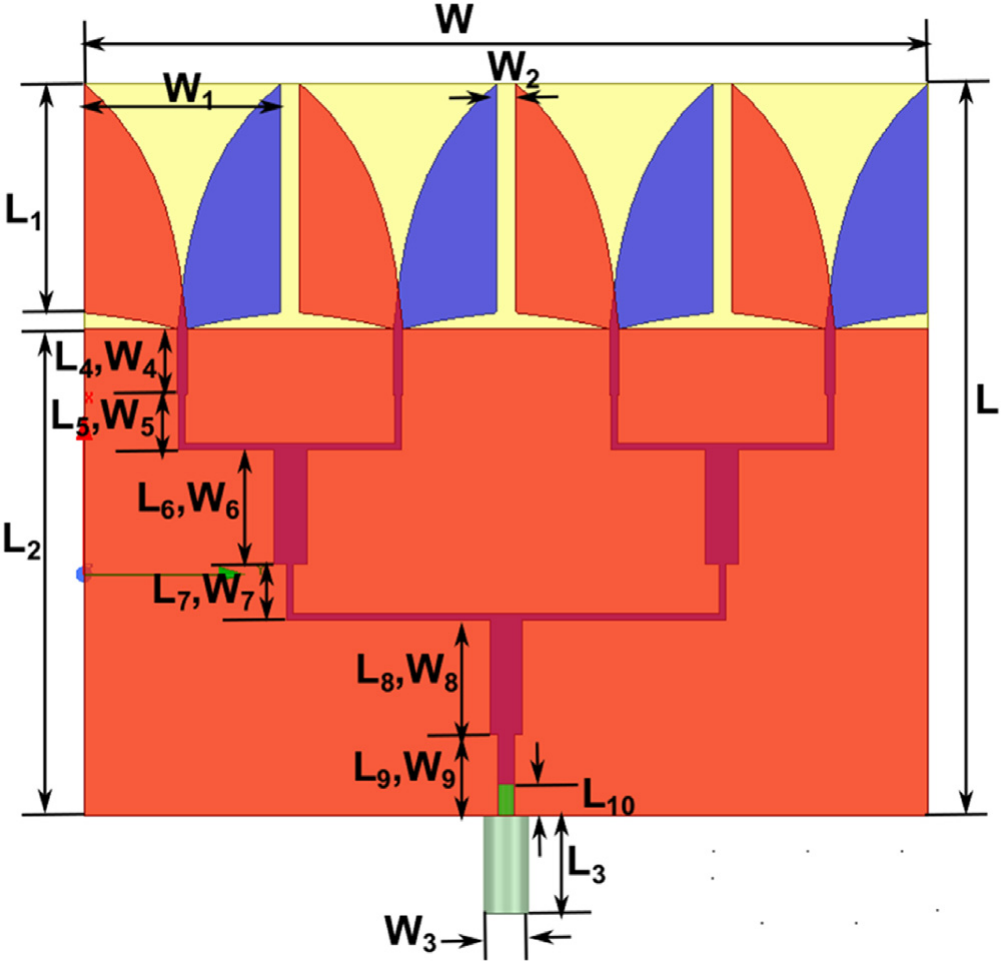
1 × 4 Conventional AVA array Design (CAVA).

Step 2: Design of 1 × 4 Conventional AVA array Design (CAVA): The second step in the design evolution is to convert a single patch to a 1 × 4 array. The 1 × 4 AVA array was designed using four single patches designed in step 1. The array configuration in an antipodal Vivaldi antenna significantly enhances performance. Fig. 3 shows a 1 × 4 Conventional AVA array Design (CAVA) design with L2 as the feeding line and W1 as the distance in the patches. In this, the blue color represents the top patch (radiator). The red color represents the bottom patch (ground). The array of elements, typically arranged in a specific geometric pattern, brings about several advantages regarding radiation characteristics, directivity, and overall antenna performance. The design dimensions of the 1 × 4 Conventional AVA array Design (CAVA) are shown in Table 2.

**Table 2:** Dimensions of AVA-PC.

Sr.No	Parameter	Measurements(mm)	Sr.No	Parameter	Measurements(mm)
1	W	25.8	13	W9	0.5
2	L	22.4	14	L1	7
3	W1	6	15	L2	14.9
4	W2	0.6	16	L3	3
5	W3	1.4	17	L4	2
6	W4	0.3	18	L5	1.5
7	W5	0.2	19	L6	3.5
8	W6	1	20	L7	1.5
9	W7	0.2	21	L8	3.5
10	W8	1	22	L9	2.5
11	L10	1	23	a	2
12	b	3.2			

**Fig. 4:**
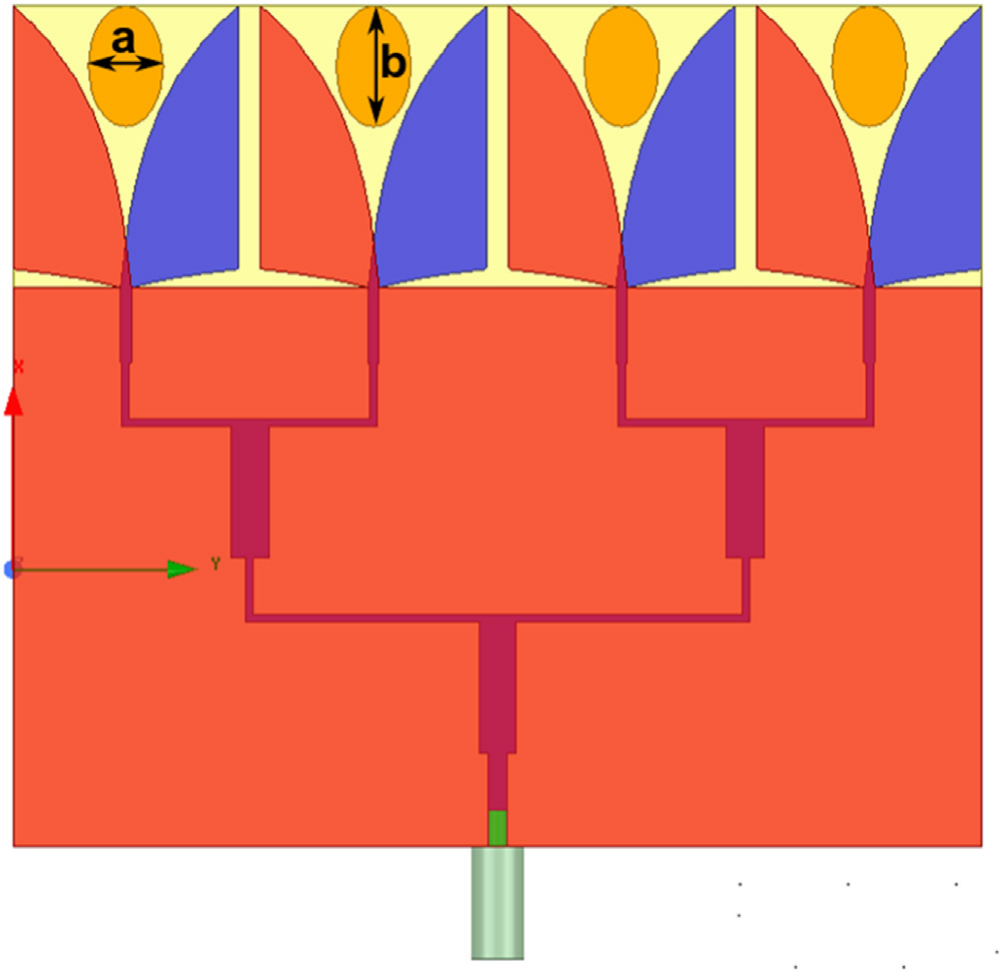
1 × 4 AVA array with parasitic patch (AVA).

Step 3: Design of 1 × 4 AVA array with parasitic patch (AVA): In the next step, a parasitic patch is added between the two flares of AVA for all elements of 1 × 4 AVA. Fig. 4 illustrates the 1 × 4 AVA array with a parasitic patch, showcasing a key performance enhancement technique. A metallic patch at the aperture end can be of different shapes, such as circular, elliptical diamond, etc. The proposed antenna employs an elliptical-shaped patch with a and b as minor and major axes. Incorporating a parasitic patch between the flares of an antipodal Vivaldi antenna contributes significantly to the overall antenna design, offering several benefits. Firstly, the parasitic patch is crucial in widening the antenna's bandwidth. While Antipodal Vivaldi antennas are renowned for their wide bandwidth, adding a parasitic patch further extends the frequency coverage, enhancing the antenna's versatility. Moreover, the parasitic patch influences the antenna's radiation pattern. Thoughtful design allows it to guide radiated energy in specific directions, improving the antenna's directivity [28], [29], [30]. This feature is valuable in applications where focused radiation or enhanced gain in particular directions is prioritized. Positioning the parasitic patch strategically contributes to increasing the antenna's gain. Through effective modification of the electromagnetic fields around the flares, the parasitic element positively influences radiation characteristics, resulting in gain improvement. The presence of the parasitic patch can also impact the antenna's impedance matching. Careful design allows the parasitic element to tune the impedance characteristics, ensuring better alignment with the transmission line and overall performance improvement [31], [32], [33], [34], [35]. Additionally, the parasitic patch assists in minimizing unwanted radiation in directions other than the main beam, reducing side lobes. This characteristic proves advantageous in applications requiring a well-defined radiation pattern with minimal interference in undesired directions. Furthermore, the parasitic patch introduces additional resonances, contributing to frequency selectivity. This proves beneficial in scenarios where the antenna needs to operate efficiently within specific frequency bands. It's important to note that the parasitic patch's design and optimization depend on the antenna's desired performance characteristics and specific application requirements. The parasitic element's dimensions, shape, and position are crucial in achieving the intended enhancements.

**Fig. 5:**
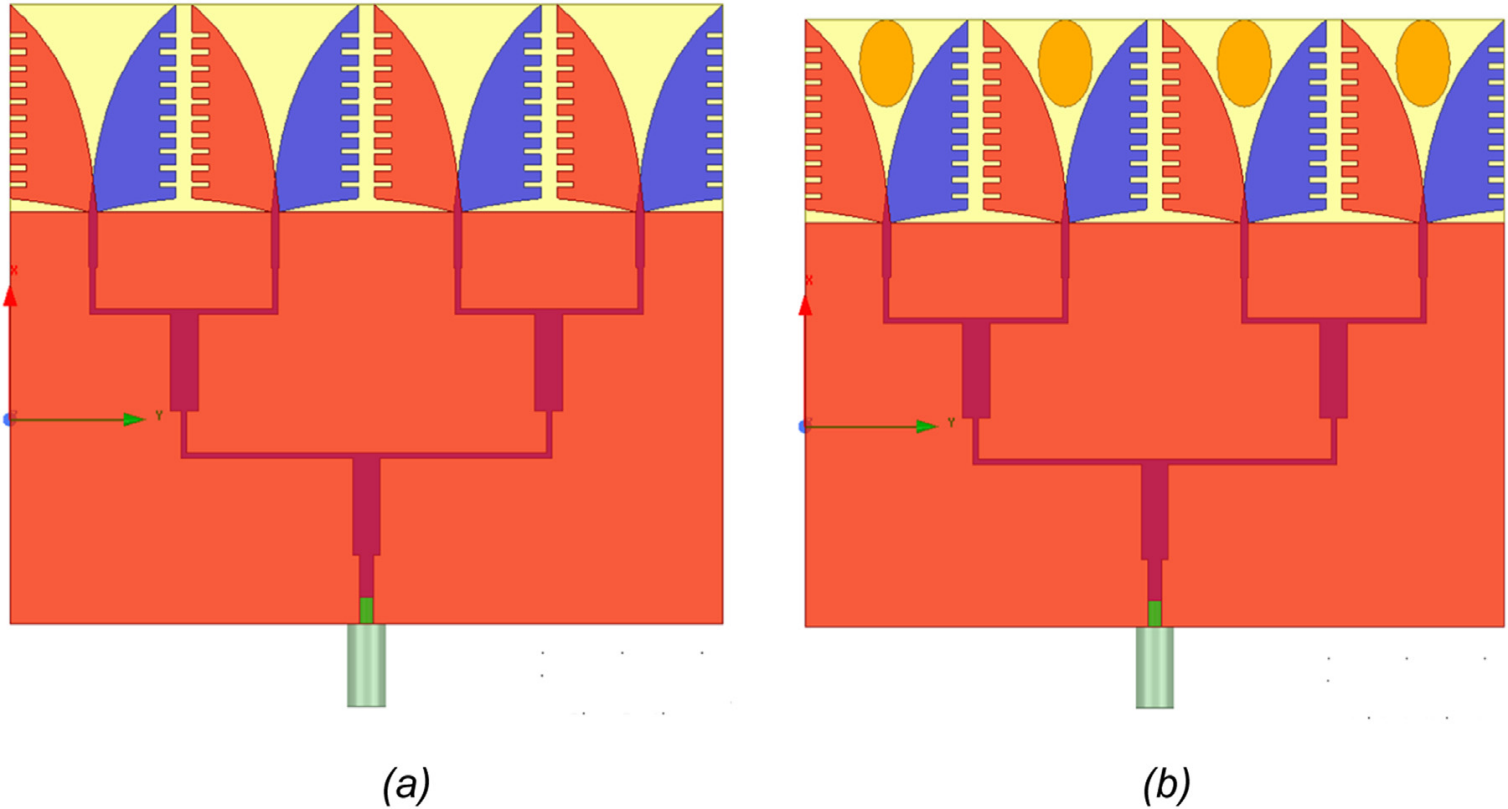
(a) 1 × 4 AVA array with corrugations (AVA-C), (b) 1 × 4 AVA array with parasitic patch and corrugation (AVA-PC).

Step 4: Design of 1 × 4 AVA array with the parasitic patch (AVA): In the fourth step of the process, the design of the 1 × 4 AVA array is enhanced by introducing corrugations to all elements' flares. Utilizing an array with corrugations in the antipodal Vivaldi antenna offers performance improvements through several mechanisms. Corrugations, characterized by periodic variations in the antenna's structure, introduce additional features that significantly influence its electromagnetic behavior. One notable contribution of corrugations is the enhancement of bandwidth. The periodic variations bring about additional resonances, enabling the antenna to operate effectively across a wider frequency range. This proves advantageous in applications requiring essential wideband operation, such as communication systems. Furthermore, the array with corrugations can lead to an increase in gain, especially in specific directions. The periodic variations in the structure contribute to constructive interference, resulting in a more focused and higher-intensity radiation pattern. This heightened gain benefits applications where directed and intensified radiation is desired. Overall, introducing corrugations to the antenna structure is pivotal in achieving performance improvements, making it suitable for various applications, including those with stringent wideband and directional requirements. This is valuable in long-distance communication systems [36]. Fig. 5(a) shows a 1 × 4 AVA array with corrugations and without a parasitic patch (AVA-C). Fig. 5(b) shows a 1 × 4 AVA array with corrugations and parasitic patches (AVA-PC).

**Fig. 6:**
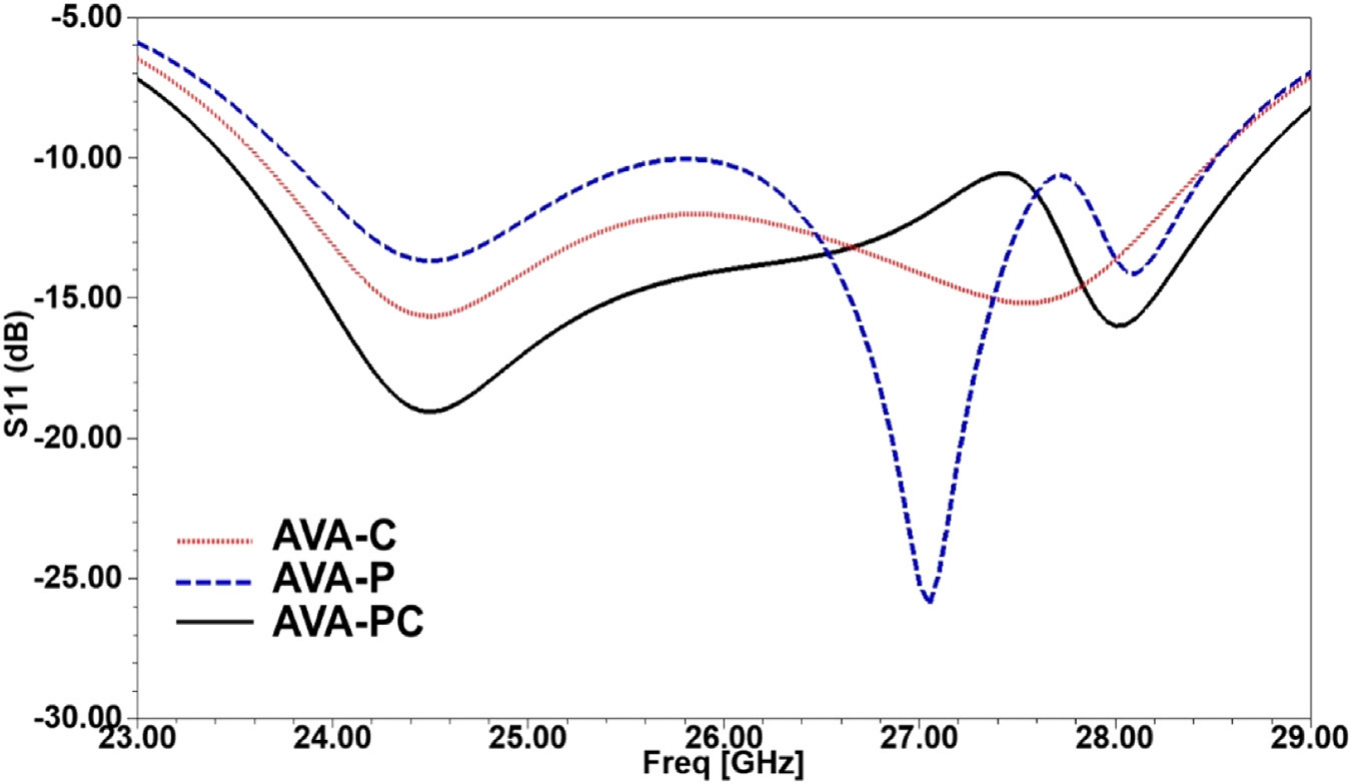
Reflection coefficients (S11(db)) versues Frequency (GHz) plot.

**Fig. 7:**
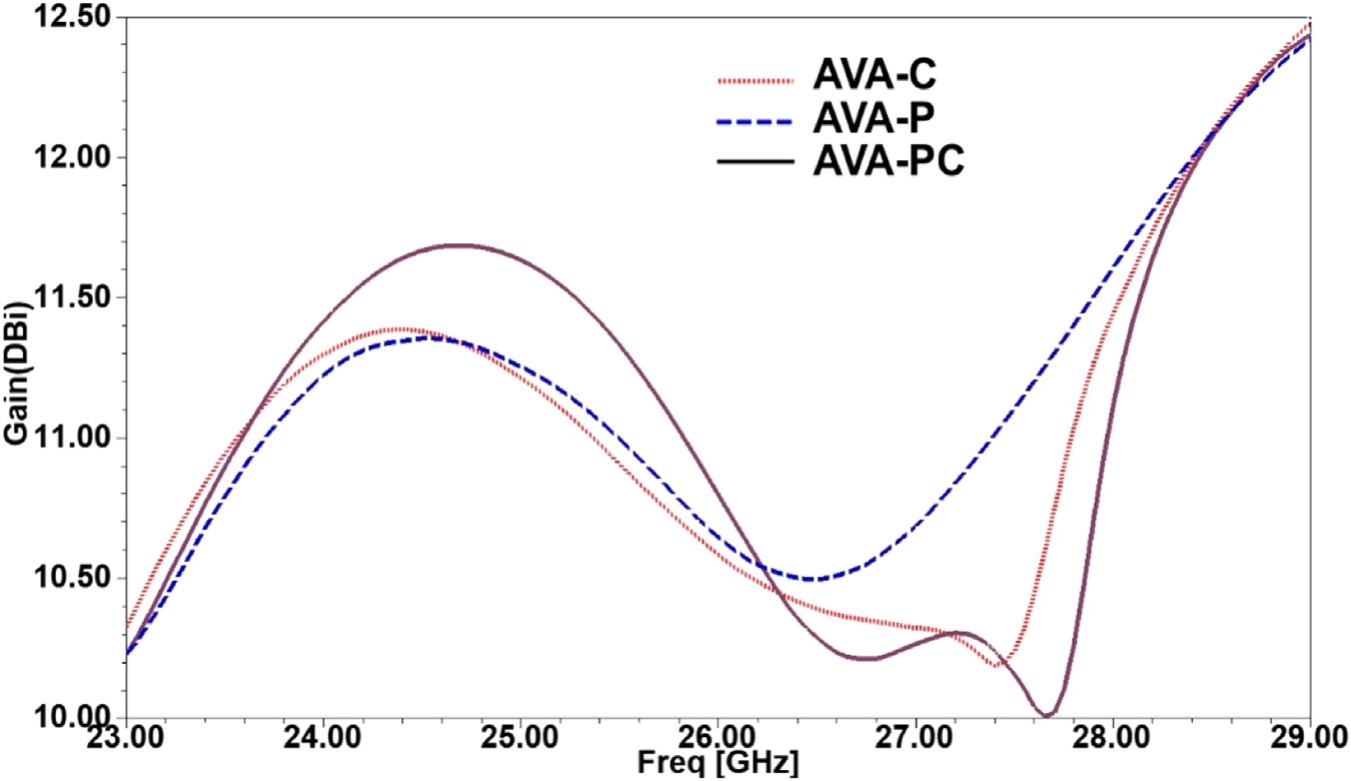
Gain(dBi) versus Frequency (GHz) plot.

The simulated outcomes of the proposed Antipodal Vivaldi Antenna (AVA) are depicted in Fig. 6, Fig. 7. In Fig. 6, the reflection coefficient (S11) is plotted w.r.t to the frequency in GHz. The reflection coefficient S11 is used in RF (Radio Frequency) engineering to quantify the signal reflected from an antenna, typically expressed in power(dBi). It is a complex number representing the ratio of the reflected signal to the incident signal at an antenna port, usually at its input. In antenna design, the ideal value of the reflection coefficient S11 is often targeted at around −10 dB or lower. All three design evolutions are plotted in the graph, i.e., AVA-C (Antipodal Vivaldi Antenna Array with Corrugation), AVA-P (Antipodal Vivaldi Antenna Array with Parasitic Patches), and AVA-PC (Antipodal Vivaldi Antenna Array with Corrugation and Parasitic Patches).

Compared to the gain range of 10.2 to 11.3 dBi for the conventional AVA, the introduced 1 × 4 AVA array, i.e., AVA-PC, achieves an enhanced gain spanning from 10 dBi to 11.7 dBi. This establishes the proposed 1 × 4 AVA array as a compelling choice for a 5 G antenna. The impact of the parasitic patch on bandwidth enhancement is clearly illustrated in Fig. 7. The blue colored line corresponds to the reflection coefficient of the AVA with the parasitic patch, while the dotted red continuous line represents the S11 of the AVA with corrugation. Featuring a major radius of 3.2 mm and a minor radius of 2 mm, the parasitic patch alters the antenna's resistance, capacitance, and inductance, influencing its frequency response. Adding the parasitic patch notably enhances the reflection coefficient of the conventional AVA. While comparing all three design evolutions, i.e., AVA-C, AVA-P, and AVA-PC, the AVA-PC is the best one with lower S11 and higher gain, which is why it is employed in the proposed antenna.

CONCLUSION: The proposed 1 × 4 Antipodal Vivaldi antenna array, equipped with a parasitic patch and corrugations, exhibits a broad operational frequency range from 23.45 GHz to 28.74 GHz. Encompassing the 28 GHz band (24.25 GHz to 29.5 GHz) crucial for 5 G applications, this antenna leverages the parasitic patch to effectively reduce the reflection coefficient below −10 dB within the frequency range from 23.45 GHz to 28.74 GHz, thereby enhancing the overall operating frequency spectrum. Additionally, the parasitic patch contributes to improved electric field radiation in the end-fire direction, elevating the gain of the Antipodal Vivaldi array (AVA). Integrating an array with corrugations further enhances performance, particularly regarding gain and bandwidth. The proposed antenna is a promising solution in advanced wireless communication with its stable gain, compact design, and suitability for 5 G applications.

## Ethics statements

This research did not involve research on humans or animals, and no data is involved from social media platforms.

## CRediT authorship contribution statement

**Sumit Kumar:** Validation, Data curation, Supervision, Funding acquisition, Project administration. **Amruta S. Dixit:** Conceptualization, Methodology, Writing – original draft, Software. **Chandan Kumar Choubey:** Writing – review & editing.

## Declaration of competing interest

The authors declare that they have no known competing financial interests or personal relationships that could have appeared to influence the work reported in this paper.

## Data Availability

No data was used for the research described in the article.
